# Duloxetine versus placebo for the treatment of women with stress predominant urinary incontinence in Taiwan: a double-blind, randomized, placebo-controlled trial

**DOI:** 10.1186/1471-2490-8-2

**Published:** 2008-01-25

**Authors:** Alex Tong-Long Lin, Mou-Jong Sun, Hui-Lung Tai, Yao Chi Chuang, Shih-Tsung Huang, Nick Wang, Yan Daniel Zhao, Julie Beyrer, Meghan Wulster-Radcliffe, Louise Levine, Curtis Chang, Lars Viktrup

**Affiliations:** 1 Division of Urology, Department of Surgery, Taipei Veterans General Hospital, Taipei, Taiwan; 2 Division of Urogynecology and Pelvic Reconstructive, Department of Obstetric and Gynecology, Changhua Christian Hospital, Changhua, Taiwan; 3 Division of Urology, Department of Surgery, Changhua Christian Hospital, Changhua, Taiwan; 4 Division of Urology, Department of Surgery, Chang Gung Memorial Hospital, Kaohsiung, Taiwan; 5 Division of Urology, Department of Surgery, Chang Gung Memorial Hospital, Lin-Kou, Taiwan; 6 Medical Department, Eli Lilly and Company, Taiwan; 7 Lilly Research Laboratories, Indianapolis, IN, USA

## Abstract

**Background:**

This manuscript compares the efficacy and safety of duloxetine with placebo in Taiwanese women with SUI.

**Methods:**

Taiwanese women with SUI were were randomly assigned to placebo (n = 61) or duloxetine 80 mg/day (n = 60) in this double-blind, 8-week, placebo-controlled study. Outcome variables included: incontinence episode frequency (IEF), Incontinence Quality of Life questionnaire (I-QOL) scores, and Patient Global Impression of Improvement rating (PGI-I).

**Results:**

Decrease in IEF was significantly greater in duloxetine-treated than placebo-treated women (69.98% vs 42.56%, P < .001). No treatment differences in I-QOL scores were significant. There were significant differences in PGI-I rating. Treatment-emergent adverse events (TEAEs) were experienced by more duloxetine-treated than placebo-treated women (80.0% vs 44.3%; P < .001). Discontinuations due to adverse events were significantly greater for duloxetine-treated than placebo-treated women (26.7% vs 6.6%; P = .003).

**Conclusion:**

Data provide evidence for the safety and efficacy of duloxetine for the treatment for Taiwanese women with SUI.

**Trial Registration:**

ClinicalTrials.gov Identifier: NCT00475358

## Background

Until recently, treatment for stress urinary incontinence (SUI), the involuntary leakage of urine on effort or exertion, or on sneezing or coughing [1], has been limited to behavioral interventions, pelvic floor muscle therapy, devices, and/or surgery [2]. In August 2004, duloxetine became the first medication approved for the treatment of women with moderate to severe SUI throughout Europe, portions of Central and South America, and the Middle East. Duloxetine is the first and only pharmaceutical agent to receive 1A rating from the International Continence Society for the treatment of SUI in women.

Duloxetine is a dual serotonin and norepinephrine reuptake inhibitor (SNRI) with little or no affinity for cholinergic receptors. Animal studies have implicated serotonin (5HT) and norepinephrine (NE) in the central control of lower urinary tract function. In cats, serotonergic agonists suppress parasympathetic activity and enhance sympathetic and somatic activity in the lower urinary tract [3-5] promoting urine storage by relaxing the bladder and increasing outlet resistance. NE variably affects the lower urinary tract depending on interactions with appropriate adrenergic receptor subtypes [6-10] The dual actions of duloxetine have been shown in the cat model to increase bladder capacity and striated urethral sphincter activity presumably through central actions in the spinal cord[11]. The ability of duloxetine to centrally stimulate pudendal motor neurons and increase striated urethral sphincter tone and contractility is believed to be the basis for its efficacy in women with SUI.

SUI is the most common type of urinary incontinence (UI) in women [12] with approximately 78% of women with UI presenting with the symptoms of SUI in either pure or mixed forms [12]. In the United States, it is estimated that 29.5 million women have SUI in a pure or mixed form. The only available studies referring to Taiwanese women suggest that the prevalence of UI in Taiwanese women (ranging from 12–44%) is lower than that of women in Western populations; however, differences in study design and criteria make it difficult to compare between studies [13-17].

Regulatory approval of duloxetine for the treatment of women with SUI in other parts of the world has been based on the demonstration of the safety and efficacy of duloxetine in 4 randomized placebo-controlled core registration trials enrolling 1913 women from Africa, Australia, Europe, and North and South America [18-21]. This study was conducted in Taiwan as a supplement to the existing core trials to comply with local regulatory requirements. The primary objective was to compare the efficacy and safety of duloxetine 80 mg/day (administered as 40 mg twice daily) with that of placebo in the treatment of Taiwanese women with predominant symptoms of SUI.

## Methods

Non-pregnant women 20 years of age and older with predominant symptoms of SUI, were enrolled in this double-blind, randomized, parallel, placebo-controlled, clinical trial conducted at 9 study centers in Taiwan. Written informed consent was obtained from all participants and study design was reviewed by a local ethics committee in accordance with the Declaration of Helsinki. Concomitant medications including urinary continence promoting drugs, antidepressants, drugs for obesity (including over-the-counter appetite suppressants and diet pills), and illicit drugs were not allowed in the study. Enrolled women reported the predominant symptoms of SUI during the last 3 months with an average of ≥ 1 incontinent episode/day. Additional history requirements included daytime voiding frequency ≤ 8 voids daily, nocturnal frequency ≤ 2 voids daily and no predominant urge incontinence symptoms. Women unable to tolerate retrograde bladder filling to 400 mL or who had a first sensation of bladder filling at ≤ 100 mL were excluded. A positive cough stress test was required after filling the bladder.

**Figure 1:**
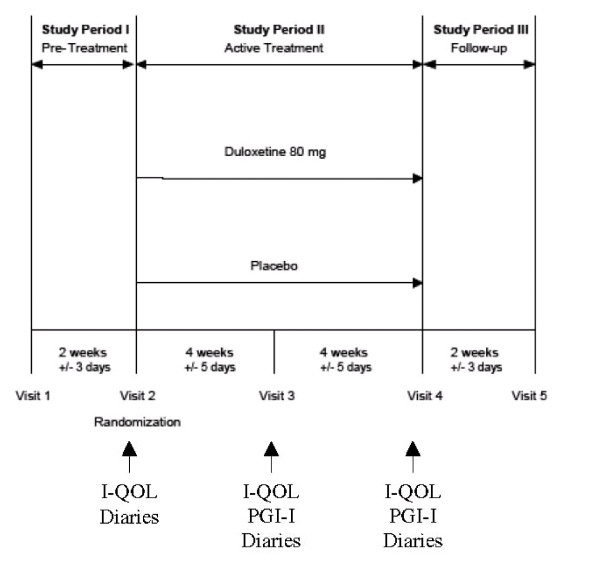
Study design and the timing of acquisition of urinary diary and quality of life measurements reported.

The study design and timing of acquisition of diaries and other variables are depicted in Figure 1. After a 2-week, no drug, lead-in period, women were randomized to 80 mg duloxetine (40 mg twice daily) or placebo for 8 weeks with post-randomization evaluation every 4 weeks. The treatment phase of the core registration trials was 12 weeks; however, the majority of the these adverse events emerged within the first 4 weeks and the number of duloxetine responders (≥ 50% reduction in median percent incontinence episode frequency [IEF]) did not change significantly after 4 weeks post-randomization. An 8-week trial was therefore considered sufficient.

Randomization was controlled by a computerized interactive voice response system at a central location for all study sites. Stratified randomization using baseline IEF of <14 or ≥14 episodes/week obtained from patient diaries was used to prevent potential imbalance in incontinence severity.

Weekly paper diaries were also used to collect the number of voids, the time of voids, and the number of continence pads used. Diaries were collected to determine baseline incontinence severity the last week before visit 2 during the no drug lead-in period (Figure 1).

The primary efficacy variable in this study was percent change in IEF/week from baseline to endpoint, which was calculated from subject completed, real-time, paper diaries. Approximately 50% reduction in IEF has been generally accepted as a clinically relevant threshold for response in SUI outcomes research for interventions including bladder training and pelvic floor muscle training [22], devices [23], surgery [24-26], and a pharmacological agent [18-21]. An IEF responder was therefore defined as a woman who had a > 50% decrease in IEF with treatment.

Secondary efficacy variables included: 1) Incontinence Quality of Life (I-QOL) questionnaire total and subscale scores [27], 2) Patient Global Impression of Improvement (PGI-I) rating[28], 3) mean time between voids/day, and 4) continence pad use/week.

The 22-item I-QOL questionnaire is a globally-validated disease-specific instrument endorsed by the International Consultation on Incontinence, which was administered at each visit. The I-QOL questionnaire evaluates the effects of UI in 3 domains: avoidance and limiting behavior, social embarrassment, and psychosocial impact. An I-QOL score of 100 represents the best possible quality of life and 0 represents the worst possible quality of life. The questionnaire has not been specifically validated in Chinese language.

The PGI-I rating is a globally-validated 1-question questionnaire and was obtained at each post-randomization visit. The PGI-I measures the improvement the subject perceives in her condition since starting treatment [28]. The questionnaire has not been specifically validated in Chinese language.

Compliance with the required study drug regiment was examined at each visit following initiation of treatment. Unused study drug was returned to Eli Lilly and Company, and compliance was assessed by counting returned study drug. Investigators encouraged compliance with study medication but subjects were not discontinued from the study for poor compliance only.

Safety was assessed by evaluation of treatment-emergent adverse events (TEAEs), discontinuations due to adverse events, serious adverse events, discontinuation emergent adverse events (DEAE), vital signs measurements, and clinical laboratory tests. Adverse events were elicited by nonprobing inquiry at each visit and were recorded regardless of perceived causality. An event was considered treatment emergent if it occurred for the first time or worsened during the double-blind treatment period. DEAE were adverse events that occurred following the end of the treatment phase and were reported during the two week follow up.

A serious adverse event was defined according to the International Consultation on Harmonization guidelines and included any adverse events associated with death, initial or prolonged inpatient hospitalization, a life-threatening experience (ie, immediate risk of dying), persistent or significant disability/incapacity, congenital anomaly/birth defect, or is significant for any other reason.

The statistical analysis plan was specified a priori and was performed in accordance with intent-to-treat (ITT) principles. Subjects with baseline and at least 1 post-baseline measurement were included in the analysis. The percent change in IEF was compared between treatment groups using the van Elteren's test, a type of stratified Wilcoxon test, with baseline incontinence severity as the stratification variable. This primary analysis compared IEF before and after randomization, pooling all diaries between visits 1 and 2 for the baseline and all diaries between visits 2 and 4 for the end point. The changes in I-QOL scores were analyzed using an ANCOVA model that included terms for baseline scores, treatment, site, and baseline incontinence severity. The endpoint PGI-I was analyzed using the Cochran-Mantel-Haenszel test with the baseline incontinence severity as the strata. The missing endpoint values in the above analyses were imputed via LOCF.

Enrollment in the study was set to end when approximately 120 women (60 per treatment group) had been assigned to a treatment group. The sample size was determined to provide 97.3% power for detecting a treatment difference of 23% in the median percent change in IEF from baseline to endpoint.

Analyses were performed using SAS 8.1 (SAS Institute, Cary, North Carolina). A two-sided alpha level of 0.05 was considered significant for treatment effects.

## Results

A total of 121 women 30–79 years of age were randomly assigned to receive duloxetine 80 mg/day (n = 60) or placebo (n = 61) between July 2003 and February 2005; overall 76.9% of women completed the 8-week study (66.7% duloxetine, 86.9% placebo).

Baseline clinical characteristics of the women randomly assigned to each treatment group were comparable (Table 1). There was a significant difference between treatment group in height (duloxetine group was shorter on average), but there were no significant differences in body mass indices (BMI) (Table 1). Additionally, women in the duloxetine treatment group voided significantly more often.

**Table 1:** Baseline^† ^clinical characteristics for all randomized women. Data are mean (SD) unless otherwise indicated

	Duloxetine	Placebo
Randomized N‡	60	61
Age, years	56.31 (± 11.00)	52.59 (± 10.25)
BMI, kg/m^2^	24.98 (± 2.96)	24.89 (± 3.13)
IEF/week (SD) [range]	15.38 (± 9.11) [5.50–47.44]	15.23 (± 8.74) [7.00–56.58]
Mean time between voids§	179.36 (± 38.81)	193.32 (± 38.00)
I-QOL score	61.88 (± 19.84)	61.21 (± 22.71)
Previous continence surgery	3	5

†Baseline is the last visit score on or prior to randomization.

‡Every randomized subject did not provide information for each variable; percentages are calculated using the number of responding women as the denominator.

§*P *= 0.048.

Abbreviations: BMI = body mass index; PFMT = pelvic floor muscle training; IEF = incontinence episode frequency; I-QOL = Incontinence Quality of Life questionnaire.

On average, patients in the placebo group took 86% of their treatment doses compared with 68% of doses for patients in the duloxetine group (*P *= .002). This difference in compliance was likely due to limited intake of duloxetine by those subjects discontinuing early from the trial and was not significant after the first post-randomization visit.

The decrease in IEF, as demonstrated by median percent change from baseline to endpoint, was significantly greater in the duloxetine group than in the placebo group (69.98% vs 42.56%, *P *< .001; Table 2). The improvements with duloxetine were observed at the first post-randomization visit (4 weeks) and were maintained throughout the study. Overall, there were significantly more IEF responders within the duloxetine-treated women than the placebo-treated women (69.6% vs 45.8%, respectively, *P *< .05).

**Table 2:** Incontinence episode frequency.

Treatment group (N)†	Time point	n‡	Median IEF/week	Median percent change from baseline	95% CI for median percent change in IEF	*P*
Placebo (61)	Baseline	59	14.00			
	Endpoint		6.25			
	Change		-5.63	-42.56	(-56.79,-33.89)	
Duloxetine (60)	Baseline	46	13.46			
	Endpoint		4.12			
	Change		-7.25	-69.98	(-81.25,-53.57)	< .001

† N = number randomized.

‡ n = number with diary data available for specified analysis.

Abbreviations: CI = confidence interval; IEF = incontinence episode frequency.

In the secondary analyses, the median percent reduction in continence pad use was numerically, but not significantly, higher for the duloxetine-treated women compared with those on placebo (-.67% vs .0%, *P *= .14). In addition to decreasing their IEF, women in the duloxetine group also numerically increased their average voiding interval compared with those in the placebo group (11.85 minutes vs .01 minutes, *P *= .13). Improvements in mean I-QOL total and subscales scores were apparent for both the duloxetine- and placebo-treated groups, but separation between the groups was not statistically significant (Tables 3 and 4). The analysis of PGI-I showed significantly more duloxetine-treated subjects than placebo-treated subjects rated their condition as "very much better" and "much better".

**Table 3:** Incontinence Quality of Life questionnaire: total score.

			I-QOL Total Score			
			
Treatment group (N)†	Time point	n‡	Mean I-QOL	Mean change in I-QOL from baseline§	95% CI for treatment difference in I-QOL	*P*
Placebo (61)	Baseline	59	61.23			
	Endpoint		74.56	13.33		
Duloxetine (60)	Baseline	52	62.65			
	Endpoint		76.29	13.64	(-4.77,6.78)	.732

†N = number randomized.

‡n = number with diary data available for specified analysis.

^§^Baseline is the last nonmissing visit score on or before randomization.

¶95% CI for treatment difference.

Abbreviations: CI = confidence interval; I-QOL = Incontinence Quality of Life questionnaire

**Table 4:** Incontinence Quality of Life questionnaire: subscale scores.

			I-QOL Avoidance and Limiting Behavior Subscale Score				I-QOL Psychological Impact Subscale Score				I-QOL Social Embarrassment Subscale Score			
			
Treatment group (N)†	Time point	n‡	Mean I-QOL	Mean change in I-QOL from baseline§	95% CI for treatment difference in I-QOL¶	*P*	Mean I-QOL	Mean change in I-QOL from baseline§	95% CI for treatment difference in I-QOL¶	*P*	Mean I-QOL	Mean change in I-QOL from baseline§	95% CI for treatment difference in IQOL¶	*P*
Placebo (61)	Baseline	59	62.34		(-5.26,6.46)		64.22		(-3.73,7.89)		54.07		(-7.37,6.82)	
	Endpoint		75.16	12.82			76.22	12.01			70.59	16.53		
Duloxetine (60)	Baseline	52	63.28				66.72				54.33			
	Endpoint		75.96	12.68		.839	79.65	12.93		.480	70.77	16.44		.940

†N = number randomized.

‡n = number with diary data available for specified analysis.

^§^Baseline is the last nonmissing visit score on or before randomization.

¶95% CI for treatment difference.

Abbreviations: CI = confidence interval; I-QOL = Incontinence Quality of Life questionnaire

TEAEs were experienced by more women in the duloxetine group compared with the placebo group (80.0% vs 44.3%; *P *< .001). Table 5 lists all of the adverse events that occurred in at least 5% of women on duloxetine or that were significantly more common with duloxetine. The very common adverse events (> 10% in either treatment group) were constipation, dry mouth, nausea, somnolence, and dizziness. Most TEAEs were reported early in the study, were mild to moderate in severity at onset, and did not increase in severity. For subjects who remained in the study despite experiencing a TEAE, the majority with fatigue (80%), nausea (100%), and somnolence (57%) had resolved within 30 days. Constipation, dry mouth, and hyperhidrosis tended to persist longer than 30 days.

**Table 5:** TEAEs occurring in ≥ 5% of women randomized to duloxetine, or significantly more often with duloxetine than with placebo.

	Duloxetine	Placebo	*P*
Values are expressed as n (%)	(N = 60)	(N = 61)	
Total number of women with ≥ 1 TEAE	48 (80)	27 (44.3)	< .001
Constipation	10 (16.7)	0 (0.0)	.001
Dry mouth	10 (16.7)	2 (3.3)	.016
Nausea	9 (15.0)	0 (0.0)	.001
Somnolence	9 (15.0)	0 (0.0)	.001
Dizziness	8 (13.3)	6 (9.8)	.583
Fatigue	5 (8.3)	0 (0.0)	.027
Hyperhidrosis	5 (8.3)	0 (0.0)	.027
Cough	4 (6.7)	4 (6.6)	> .999
Decreased appetite	4 (6.7)	1 (1.6)	.207
Insomnia	4 (6.7)	2 (3.3)	.439
Asthenia	3 (5.0)	1 (1.6)	.365
Chest discomfort	3 (5.0)	3 (4.9)	> .999
Palpations	3 (5.0)	2 (3.3)	.680

The discontinuation rate due to adverse events was significantly greater for the duloxetine group compared with the placebo group (26.7% vs 6.6%; *P *= .003). Treatment differences were not significant for any single event. The most common adverse event leading to discontinuation (≥5% in the duloxetine treatment group) was dizziness. Table 6 lists all adverse events that resulted in a >1% discontinuation rate for duloxetine.

**Table 6:** Discontinuations for adverse events in >1% women randomized to duloxetine. Values are expressed as n (%).

	Duloxetine	Placebo	*P*
	(N = 60)	(N = 61)	

For any adverse event	16 (26.7)	4 (6.6)	.003
Dizziness	4 (6.7)	2 (3.3)	.439
Nausea	2 (3.3)	0 (0.0)	.244
Somnolence	2 (3.3)	0 (0.0)	.244
Asthenia	1 (1.7)	0 (0.0)	.496
Flatulence	1 (1.7)	0 (0.0)	.496
Hypertension	1 (1.7)	0 (0.0)	.496
Insomnia	1 (1.7)	0 (0.0)	.496
Palpitations	1 (1.7)	0 (0.0)	.496
Phobia	1 (1.7)	0 (0.0)	.496
Urinary hesitation	1 (1.7)	0 (0.0)	.496
Vomiting	1 (1.7)	0 (0.0)	.496

Clinical laboratory assessments, vital signs, and physical findings were stable relative to baseline and no clinically relevant differences were detected between treatment groups. A significant difference between treatment groups was observed in the mean change in heart rate; however, the 2.57 beat per minute (bpm) increase with duloxetine was not considered clinically important. There was also a significant 4.12 mmHg difference in the baseline to endpoint diastolic blood pressure between treatment groups, but the 2.57 mmHg mean increase with duloxetine was not considered clinically important.

Four duloxetine-treated subjects experienced 4 DEAEs, while, placebo-treated subjects experienced 13 DEAEs. Four subjects (1 duloxetine-treated and 3 placebo-treated) experienced serious adverse events (SAEs). The reported SAE in 1 duloxetine-treated subject was coronary artery disease; the 3 SAEs reported in placebo-treated subjects were eye injury, calculus ureteric, and a urinary tract infection. There were no deaths in this study.

## Discussion

In this study of Taiwanese women with predominant SUI, duloxetine 80 mg/day (40 mg twice daily) as measured by the primary efficacy analysis (median percent change in IEF/week) was noted to be significantly more effective than placebo. Duloxetine was significantly superior to placebo as measured by PGI-I responses, but there were no differences between treatment groups for I-QOL total or subscale scores. The significant reductions in IEF episodes and in PGI-I with duloxetine compared with placebo in this trial, are consistent with responses from previously published core registration trials conducted in Africa, Australia, Europe, and North and South America [18-21].

Previous studies have shown that women with SUI begin to appreciate that their condition has improved with treatment when they reduce their incontinence by half [28]. There was a 50–100% reduction in incontinence episodes in 69.6% of the Taiwanese women treated with duloxetine. In fact, the treatment difference of 23.8% for IEF responders was somewhat greater than the treatment differences observed in the analysis of the integrated database from the 4 core registration trials [18-21]. Consistent with the core registration trials, the significant duloxetine group improvements in IEF were apparent within the first 4 weeks of treatment and were maintained throughout the duration of the 8-week study.

Incontinence improved despite numerical but not statistically significant increases in the time between voids with duloxetine, indicating the improvement did not result from more frequent emptying of the bladder.

Although, I-QOL total and subscale scores improved in women with both duloxetine and placebo-treated women from baseline, the differences were not significant. The overall improvement, but lack of separation between treatments, despite significant treatment differences on multiple other measures of efficacy, suggests that this quality-of-life instrument may not be reflective of improvements in incontinence either in its Chinese translation or in Taiwanese women. UI is often cited as a psychologically distressing, socially secluding and potentially disabling condition. In Western countries the impact of UI on quality of life is comparable to the impact of diabetes on quality of life [29,30]. A single study of Taiwanese women suggests a greater impact on quality of life in Taiwanese women than in other cultures [14] and attributed this to more stringent behavioral modifications in Taiwan [14]. Nevertheless, the baseline, incontinence-specific quality of life in these Taiwanese women, as measured by I-QOL, was similar to that of Western women described in an integrated analysis of 1913 women [18-21].

Significantly more duloxetine-treated women than placebo-treated women experienced TEAEs with 26.7% of duloxetine-treated women discontinuing therapy due to adverse events. Unlike the core registration studies, this study did not have a 2-week placebo lead-in phase which may have increased the number of TEAEs reported relative to those studies in which the discontinuation rate due to adverse events was 20.5% for duloxetine-treated subjects. Mean BMI in the Taiwanese women was lower than the mean BMI in women included in the core studies which could contribute to the increased number of women reporting TEAEs and discontinuing.

Nausea was a frequent adverse event associated with duloxetine treatment; 3.3% of all duloxetine patients discontinued due to nausea, although the majority of women who experienced duloxetine-related nausea completed the study. Nausea tended to start soon after the initiation of duloxetine treatment. In most cases, nausea was mild to moderate, did not worsen after its onset, and resolved within 1 week to 1 month of therapy.

Overall, the majority of TEAEs reported by > 5% of duloxetine-treated subjects and significantly more often than in placebo-treated subjects, tended to be mild in severity. One subject reported severe nausea. In no instance did any of these TEAEs increase in severity. The majority of women that experienced fatigue, nausea, and somnolence but remained in the study had resolution of the events within 30 days.

Constipation, dry mouth, and hyperhidrosis tended to persist longer than 30 days. This TEAE profile is largely consistent with published data from Africa, Australia, Europe, and North and South America [18-21]. The improvement associated with duloxetine treatment should be weighed against a considerable discontinuation rate due to early adverse events.

## Conclusion

The data from this trial support the conclusion that duloxetine has demonstrated similar efficacy and safety in Taiwanese women with SUI as has been demonstrated in women in Africa, Australia, Europe, and North and South America. Duloxetine administered at 40 mg twice daily for up to 8 weeks for the treatment of Taiwanese women with SUI is safe and efficacious. Finally, the data also support the conclusion that the findings from studies in other populations, with the exception of I-QOL, can be reasonably extrapolated to the Taiwanese population.

## Competing interests

Alex Tong-Long Lin, Mou-Jong Sun, Hui-Lung Tai, Yao Chi Chuang, and Shih-Tsung Huang declare that they have no competing interests.

Nick Wang, Yan Daniel Zhao, Curtis Chang, Lars Viktrup and Julie Beyrer are employees of Eli Lilly and Company and receive a salary from Eli Lilly and associated stock options in the company.

Meghan Wulster-Radcliff eand Louise Levine are previous employees of Eli Lilly and Company and received a salary from Eli Lilly and associated stock options in the company.

## Authors' contributions

ATLL: Made substantial contributions to acquisition and interpretation of data, revised the article critically for important intellectual content, read and approved the final manuscript.

MJS: Made substantial contributions to acquisition interpretation of data, revised the article critically for important intellectual content, read and approved the final manuscript.

HLT: Made substantial contributions to acquisition and interpretation of data, read and approved the final manuscript.

YCC: Made substantial contributions to acquisition and interpretation of data, read and approved the final manuscript.

STH: Made substantial contributions to acquisition of data, and interpretation of data, read and approved the final manuscript.

NW: Was involved in the design of the study, acquisition of data and drafting and reviewing the manuscript including reading and giving final approval of the manuscript.

YDZ: Was involved in the design of the study and all analysis of the data as well as drafting the manuscript and reading and providing final approval of the manuscript.

JB: Was involved in the design of the study and drafting and reviewing the manuscript including reading and giving final approval of the manuscript.

MWR: The author was involved in drafting and reviewing the manuscript including reading and giving final approval of the manuscript.

LL: The author was involved in the design of the study, acquisition of data and drafting and reviewing the manuscript including reading and giving final approval of the manuscript.

CC: The author was involved in the design of the study, acquisition of data and drafting and reviewing the manuscript including reading and giving final approval of the manuscript.

LV: The author was involved in the design of the study, acquisition of data and drafting and reviewing the manuscript including reading and giving final approval of the manuscript.

## Pre-publication history

The pre-publication history for this paper can be accessed here:


